# Association of transitions in frailty with dementia risk: findings from two longitudinal cohort studies

**DOI:** 10.3389/fmed.2026.1782916

**Published:** 2026-03-26

**Authors:** Mingrui Liu, Zelin Ye, Haiyu Liu, Pengzhen Ma, Yaning Li, Qihao Wang, Yikang Shen, Huaxin Pang, Xiaoxia Xie, Yufeng Zhao

**Affiliations:** 1 Data Center of Traditional Chinese Medicine, China Academy of Chinese Medical Sciences, Beijing, China; 2 Department of Infection, Guang’anmen Hospital, China Academy of Chinese Medical Sciences, Beijing, China

**Keywords:** aging population, chronic disease burden, dementia, frailty, incidence

## Abstract

**Introduction:**

Previous studies have suggested that frailty is an important risk factor for dementia. Frailty is a dynamic condition that may progress or improve. However, the impact of frailty transitions on dementia risk has not been sufficiently examined.

**Methods:**

We analyzed data from two prospective cohorts. Frailty status was assessed using the Frailty Index (FI) and categorized into three groups: robust, pre-frail, and frail. Transitions in frailty status were evaluated based on FI at baseline and at a second assessment. The outcome was dementia, which was ascertained by self-reported physician diagnosis. To adjust for potential confounders, a directed acyclic graph was drawn, and Cox proportional hazards models were used to calculate hazard ratio (HR) and 95% confidence interval (95% CI).

**Results:**

Based on the inclusion and exclusion criteria, a total of 8,353 participants were included in the analysis of transitions in frailty status. Compared with stable robust participants, robust participants who progressed to pre-frail or frail had an increased risk of dementia (HR = 1.78, 95% CI: 1.18–2.68, *p* = 0.006). Compared with stable pre-frail participants, pre-frail participants who progressed to frail also had a significantly higher risk of dementia (HR = 1.97, 95% CI: 1.39–2.81, *p* < 0.001). However, the difference in dementia risk between pre-frail participants who reverted to robust status and stable pre-frail was not statistically significant (HR = 1.24, 95% CI: 0.80–1.92, *p* = 0.342). Similarly, the difference in dementia risk between frail participants who improved to pre-frail or robust status and stable frail was not statistically significant (HR = 0.94, 95% CI: 0.62–1.41, *p* = 0.754). The results of the sensitivity analyses were generally consistent with those of the main analysis, supporting the robustness of the findings.

**Conclusion:**

Progression in frailty status was associated with an increased risk of dementia compared with stable robust or stable pre-frail status, whereas recovery from pre-frail or frail status was not associated with a significant reduction in dementia risk.

## 1 Introduction

Dementia is a chronic and progressive syndrome characterized by a decline in cognitive function that is more severe than that in normal aging (1). Due to population aging and the absence of effective cures, the global number of dementia cases is projected to rise from 55 million in 2019 to 139 million by 2050 (2). Currently, there is no pharmacological treatment capable of altering the progression of dementia. Drugs approved by the United States Food and Drug Administration only provide symptomatic relief and are associated with serious adverse events such as stroke and death (3), highlighting the urgent need for safe, cost-effective strategies to reduce risk and delay onset. Although advancing age is widely recognized as a primary risk factor for dementia (4), the likelihood of developing dementia varies considerably among individuals of the same age group (5).

Emerging evidence indicates that an individual’s overall health status, particularly the accumulation of multiple health deficits, may offer a more informative perspective on dementia risk, with this multidimensional vulnerability often described as frailty (6–8). Frailty is characterized by heightened vulnerability to minor stressors, diminished physiological reserve, and its association with adverse health outcomes, including disability, hospitalization, falls, and mortality (9, 10). Increasing evidence indicates that frailty is also closely linked to cognitive outcomes. Higher frailty index (FI) scores have been associated with increased risk of cognitive impairment in older adults (11). In long-term care settings, greater accumulation of health deficits has been associated with increased risks of dementia and mortality (12). In the UK Biobank cohort of 274,194 participants, physical frailty was prospectively associated with incident dementia, with hazard ratios of 1.396 for pre-frailty and 2.304 for frailty compared with non-frailty (6). In addition, a study based on the English Longitudinal Study of Aging (ELSA) followed 8,722 participants free of dementia at baseline and reported that 365 individuals (4.2%) developed dementia during follow-up, with higher risks observed among those classified as pre-frail or frail compared with non-frail participants (5). The underlying biological mechanisms linking frailty to cognitive impairment and dementia are complex and may involve a range of shared physiological, functional, and social pathways, such as poor vascular health (13), chronic systemic inflammation (14, 15), oxidative stress (16), and social isolation (17).

Frailty is not a static condition. Numerous studies have demonstrated that frailty is a dynamic condition that can both progress and improve over time (18, 19). However, most prior investigations have focused on frailty measured at a single time point. Evidence regarding whether transitions in frailty status are associated with subsequent cognitive outcomes remains limited. A study among older adults in South Korea reported that individuals who remained frail or transitioned to frail status experienced greater cognitive decline over time (20). However, evidence linking transitions in frailty status to incident dementia remains limited.

The Health and Retirement Study (HRS) and ELSA are nationally representative longitudinal surveys of adults aged 50 years and older in the United States and England, respectively. Both studies were designed to provide interdisciplinary data on aging, including health status, functional limitations, cognitive performance, socioeconomic position, family structure, health care utilization, and retirement (21, 22). ELSA was modeled after the HRS, and harmonized data files have been developed to standardize key variables across cohorts. Integrating data from these two cohorts increases sample size, enhances statistical power, and improves the precision and stability of the estimated associations. Building on this framework, the present study examines how dynamic transitions in frailty are associated with dementia risk using integrated data from ELSA and HRS. Frailty status is assessed using the FI. We hypothesize that progression in frailty is associated with an increased risk of dementia, whereas improvement in frailty is associated with a reduced risk.

## 2 Methods

This study was conducted following the Strengthening the Reporting of Observational Studies in Epidemiology (STROBE) reporting guidelines (23) (Supplementary Table S1).

### 2.1 Study design and population

ELSA and HRS were initiated in 2002 and 1992, respectively, and participants in both studies have been followed up every 2 years. Ethical approval for the ELSA and HRS studies was obtained from the London Multi-Centre Research Ethics Committee and the University of Michigan Institutional Review Board, respectively. All participants signed the informed agreement to participate.

Participants eligible for this study were adults aged 50 years and older, consistent with the design of ELSA and HRS. In this study, we integrated data from ELSA and HRS. Wave 5 of ELSA (2010–2011) and Wave 10 of HRS (2010) were defined as the baseline. Wave 6 of ELSA (2012–2013) and Wave 11 of HRS (2012) were defined as the second assessment. Baseline frailty status was evaluated using baseline data, while dynamic transitions in frailty status were evaluated using data from both baseline and the second assessments. To ensure consistency in the follow-up period between cohorts, Wave 9 of ELSA (2018–2019) and Wave 14 of HRS (2018) were used as the endpoint of follow-up.

**Figure 1:**
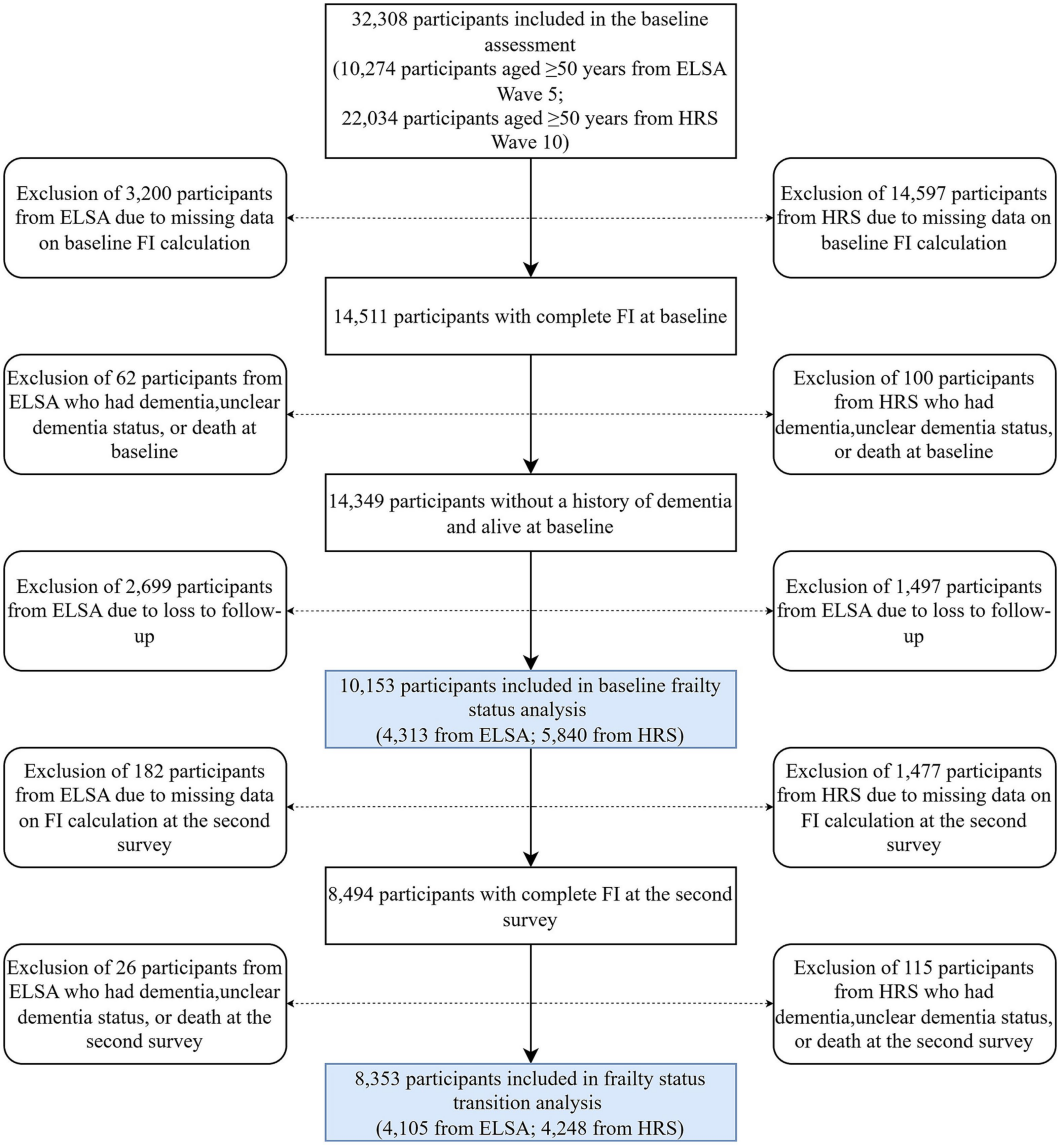
Flow diagram of the study population selection process. ELSA, English Longitudinal Study of Aging; HRS, Health and Retirement Study; FI, frailty index.

Among the 32,308 participants from ELSA and HRS, 17,797 were excluded due to missing data for baseline FI calculation. A further 162 were excluded due to dementia diagnosis, unclear dementia status, or death at baseline. An additional 4,196 were excluded due to loss to follow-up. As a result, 10,153 participants (4,313 from ELSA and 5,840 from HRS) were included in the baseline frailty status analysis. Subsequently, 1,659 were excluded due to missing data for FI calculation at the second assessment, and 141 were excluded due to dementia diagnosis, unclear dementia status, or death at the second assessment. Since participants lost to follow-up had already been excluded, 8,353 (4,105 from ELSA and 4,248 from HRS) were included in the analysis of transitions in frailty status. The selection process of the study population is shown in Figure 1.

### 2.2 Assessment of frailty

Frailty was measured using the FI, a validated and widely adopted approach in epidemiological research (9). The FI was constructed following the standard procedure described earlier (24, 25). The FI includes 35 variables encompassing a range of domains, including chronic conditions (excluding dementia), symptoms, disabilities, self-rated health, physical function, and depressive symptoms (Supplementary Table S2). Each variable contributed equally to the FI, with a value of 1 assigned if a deficit was present and 0 if absent. The FI was calculated by dividing the number of deficits present by the total number of variables (35). As in previous studies, frailty status was categorized into three groups based on FI thresholds: robust (FI ≤ 0.10), pre-frail (0.10 < FI < 0.25), and frail (FI ≥ 0.25) (26, 27). Transitions in frailty status were determined by comparing FI values at baseline and the second assessments. To further capture the dynamic transitions of frailty, we additionally assessed the total FI score—calculated as the sum of FI at baseline and at the second assessment—and the change in FI score, calculated as the difference between the second and baseline FI values.

### 2.3 Outcome and follow-up

In this study, the primary outcome was dementia. Dementia was identified based on self-reported physician diagnosis. At each wave of ELSA and HRS, participants were asked the question: “whether a doctor has told the respondent they have dementia, organic brain senility, or any other serious memory condition.” Individuals self-reporting a diagnosis were defined as dementia cases.

For both analytical frameworks, the baseline wave (Wave 5 of ELSA and Wave 10 of HRS) was defined as the initial exposure assessment time point. For the baseline frailty analysis, frailty status was determined at the baseline wave, and follow-up for incident dementia began at the subsequent wave (Wave 6 of ELSA and Wave 11 of HRS) to ensure that exposure preceded outcome occurrence. For the analysis of transitions in frailty status, frailty transitions were defined based on frailty status at the baseline and second assessment waves (Wave 5 and Wave 6 in ELSA; Wave 10 and Wave 11 in HRS). To maintain correct temporal ordering, follow-up for incident dementia began at the wave following the second assessment (Wave 7 of ELSA and Wave 12 of HRS). In both analyses, participants were followed until dementia diagnosis, death, or the end of follow-up (Wave 9 of ELSA and Wave 14 of HRS), whichever occurred first. Death data in HRS were available up to the most recent wave, while for ELSA, information on death was only accessible up to Wave 6 (2012–2013) (28).

### 2.4 Covariates

To identify potential confounders, we applied a directed acyclic graph (DAG) based on established epidemiological guidelines (29). First, we conducted a comprehensive literature review to identify variables potentially associated with both FI and dementia. Second, the selected variables were structured within a theoretical framework according to their temporal order and presumed causal pathways. Third, we utilized the DAGitty web application^1^ to construct and visualize the causal diagram. Based on the minimally sufficient adjustment sets identified through DAG analysis, the final models included the following covariates: age, gender, education level, marital status, smoking, drinking, social activities, and body mass index (BMI). To ensure consistency in covariate definitions between ELSA and HRS, marital status was categorized as either married or partnered or not married (including separated, divorced, widowed, or never married). Education level was categorized into three levels: less than high school, high school, and college or higher education. Smoking was grouped into never and ever smokers. Drinking was classified as never and ever drinkers. Social activities were classified as engaged or not engaged. A visual representation of the DAG is shown in Supplementary Figure S1.

### 2.5 Statistical analysis

Baseline characteristics were presented according to FI categories, with continuous variables expressed as mean [standard deviation (SD)] and categorical variables as number (percentage).

To examine the relationship between baseline frailty status and the risk of dementia, Cox proportional hazards models were applied to estimate hazard ratios (HRs) and their corresponding 95% confidence intervals (CIs). Robust individuals served as the reference group, and two models were fitted. Model 1 included no covariate adjustment, while Model 2 was adjusted for potential confounders, including age, gender, education level, marital status, smoking, drinking, social activities, and BMI. Missing values for covariates were imputed through multiple imputation using chained equations. Multiple imputation was conducted using the “mice” package in R. Following recommendations from previous studies, only covariates with less than 80% missingness were imputed (30). Ten imputed datasets were generated, and effect estimates were calculated separately and then pooled using Rubin’s rules (31). The percentage of missing data for each variable is reported in Supplementary Tables S3, S4. Using similar modeling strategies, we further explored how transitions in frailty status, total FI score, and change in FI score were related to dementia risk. Both total FI score and change in FI score were categorized into tertiles, using the lowest tertile as the reference category. In analyses involving change in FI score, baseline FI was additionally adjusted for in the multivariable model. Trend tests were performed by treating the tertile variable as a continuous term in the model. To evaluate the proportional hazards assumption, Schoenfeld residuals were examined (32). A global test of Schoenfeld residuals was conducted to detect potential violations for any of the covariates. For variables that showed potential violations, time-dependent covariates were introduced to address this issue (33).

We performed several sensitivity analyses to evaluate the stability of our findings: (i) we conducted sensitivity analyses on transitions in frailty status by further accounting for baseline FI in the adjustment model; (ii) to ensure that observed frailty transitions were not transient, we re-assessed frailty status during the third wave; (iii) to reduce potential reverse causation, we repeated the primary analyses after excluding participants diagnosed with dementia during the first year of follow-up; (iv) because the optimal FI thresholds for classifying frailty status remain debated (34), we applied two additional commonly used thresholds for sensitivity analysis. In cut-off 1, frailty categories were defined as: frail (FI > 0.21), pre-frail (0.10 < FI ≤ 0.21), and robust (FI ≤ 0.10) (35). In cut-off 2, frailty categories were defined as: frail (FI ≥ 0.25), pre-frail (0.08 < FI < 0.25), and robust (FI ≤ 0.08) (5); (v) considering death as a competing event for dementia, we applied a competing risk model to repeat the main analyses; (vi) sex-stratified analyses were conducted, and interactions were tested using likelihood ratio tests; (vii) because of the limited number of dementia events among participants aged <65 years, a sensitivity analysis was performed in those aged ≥65 years; (viii) To examine whether the observed associations were consistent within each cohort, we conducted cohort-specific analyses by repeating all primary models separately in the HRS and ELSA datasets.

All statistical analyses were performed using R (version 4.4.2). A *p* < 0.05 (two-tailed) was considered indicative of statistical significance.

## 3 Results

### 3.1 Baseline characteristics

According to the predefined inclusion and exclusion criteria, 10,153 participants (female: 55.85%, mean age: 77.07 years) from the integrated ELSA and HRS cohorts were included in the baseline frailty status analysis. Baseline characteristics of the study population are summarized in Table 1. Participants classified as frail are generally older, more likely to be female, and show comparable distributions of marital status across frailty groups, with similar proportions being married or partnered versus other categories. Relative to robust participants, those classified as frail exhibited lower levels of education and elevated BMI. For the analyses of transitions in frailty status, 8,353 participants (female: 55.55%, mean age: 71.06 years) met the criteria. Their baseline characteristics are summarized in Table 2. Frail participants in this analysis are generally older, predominantly female, and have a higher likelihood of being married or partnered. Compared with robust participants, they also tend to have reduced educational levels and increased body mass index. Additionally, we summarized the baseline characteristics using the non-imputed dataset (Supplementary Tables S5, S6). The findings were comparable to those reported in Tables 1, 2.

**Table 1:** Baseline characteristics of participants for baseline frailty status analyses.

Variables	ELSA and HRS (*n* = 10,153)		
	Robust	Pre-frail	Frail
Number (%)	4,027	3,683	2,443
Age, mean (SD), years	69.56(6.49)	72.77(6.75)	75.16(7.69)
Sex, n (%)			
Male	2,056(51.06)	1,552(42.14)	875(35.82)
Female	1,971(48.94)	2,131(57.86)	1,568(64.18)
Marital status, n (%)			
Married or partnered	2,970(73.75)	2,453(66.60)	1,254(51.33)
Other marital status	1,057(26.25)	1,230(33.40)	1,189(48.67)
Education level, n (%)			
Below high school	895(22.23)	841(22.83)	868(35.53)
High school	1,102(27.37)	1,206(32.75)	816(33.40)
College or above	2,030(50.41)	1,636(44.42)	759(31.07)
Smoking, n (%)			
Never smokers	1,724(42.81)	1,488(40.40)	885(36.23)
Ever smokers	2,303(57.19)	2,195(59.60)	1,558(63.77)
Drinking, n (%)			
Never drinkers	801(19.89)	1,246(33.83)	1,312(53.70)
Ever drinkers	3,226(80.11)	2,437(66.17)	1,131(46.30)
Social activities, n (%)			
Yes	2,217(55.05)	2,204(59.84)	1,387(56.77)
No	1,810(44.95)	1,479(40.16)	1,056(43.23)
BMI, mean (SD), kg/m^2^	27.75(4.77)	28.95(5.33)	29.51(5.82)

ELSA, English Longitudinal Study of Aging; HRS, Health and Retirement Study; BMI, body mass index.

**Table 2:** Baseline characteristics of participants for transitions in frailty status analyses.

Variables	ELSA and HRS (*n* = 8,353)		
	Robust	Pre-frail	Frail
Number (%)	3,692	3,045	1,616
Age, mean (SD), years	69.15(6.22)	72.08(6.46)	73.50(7.08)
Sex, n (%)			
Male	1,881(50.95)	1,279(42.00)	553(34.22)
Female	1,811(49.05)	1,766(58.00)	1,063(65.78)
Marital status, n (%)			
Married or partnered	2,754(74.59)	2,071(68.01)	885(54.76)
Other marital status	938(25.41)	974(31.99)	731(45.24)
Education level, n (%)			
Below high school	821(22.24)	673(22.10)	557(34.47)
High school	980(26.54)	972(31.92)	524(32.43)
College or above	1,891(51.22)	1,400(45.98)	535(33.11)
Smoking, n (%)			
Never smokers	1,582(42.85)	1,220(40.07)	591(36.57)
Ever smokers	2,110(57.15)	1,825(59.93)	1,025(63.43)
Drinking, n (%)			
Never drinkers	657(17.80)	912(29.95)	773(47.83)
Ever drinkers	3,035(82.20)	2,133(70.05)	843(52.17)
Social activities, n (%)			
Yes	2,000(54.17)	1,772(58.19)	895(55.38)
No	1,692(45.83)	1,273(41.81)	721(44.62)
BMI, mean (SD), kg/m^2^	27.77(4.73)	29.23(5.41)	29.99(5.92)

ELSA, English Longitudinal Study of Aging; HRS, Health and Retirement Study; BMI, body mass index.

In the baseline frailty status analysis, the median follow-up time was 8.0 years, with 2,259 deaths occurring during the follow-up period. In the transitions in frailty status analysis, the median follow-up time was also 8.0 years, with 1,191 deaths occurring during the follow-up period.

### 3.2 Association of baseline frailty status with dementia

The relationship between baseline frailty status and dementia risk is presented in Supplementary Table S7. After adjusting for potential confounders, Model 2 showed that participants classified as frail were at a significantly elevated risk of dementia compared to those who were robust (HR 2.61, 95% CI 2.11–3.24), and pre-frail participants also showed a significantly elevated risk (HR 1.51, 95% CI 1.23–1.86).

### 3.3 Association of transitions in frailty status with dementia

Table 3 displays the number and proportion of frailty status transitions observed over the two-year follow-up period. Among those classified as robust at baseline, 764 participants (20.69%) from ELSA and HRS progressed to pre-frail or frail status. Among participants who were pre-frail at baseline, 522 (17.14%) improved to robust status, while 502 (16.49%) progressed to frail status. Meanwhile, among those classified as frail at baseline, 306 participants (18.94%) improved to pre-frail or robust status. Table 4 presents the relationship between transitions in frailty status and the risk of dementia. Relative to stable robust participants, those who progressed to pre-frail or frail status exhibited a significantly higher risk of dementia (HR 1.78, 95% CI 1.18–2.68). In contrast, relative to stable frail participants, those who improved to pre-frail or robust status did not have a statistically significant difference in dementia risk (HR 0.94, 95% CI 0.62–1.41). Among stable pre-frail participants, those who progressed to frail status had a significantly increased risk of dementia (HR 1.97, 95% CI 1.39–2.81). However, relative to stable pre-frail participants, those who improved to robust status did not exhibit a statistically significant difference in dementia risk (HR 1.24, 95% CI 0.80–1.92).

**Table 3:** Number and proportion of the transitions in frailty status.

Baseline	The second survey	ELSA and HRS n (%)
Robust	Robust	2,928(79.31)
	Pre-frail	714(19.34)
	Frail	50(1.35)
Pre-frail	Robust	522(17.14)
	Pre-frail	2021(66.37)
	Frail	502(16.49)
Frail	Robust	19(1.18)
	Pre-frail	287(17.76)
	Frail	1,310(81.06)

ELSA, English Longitudinal Study of Aging; HRS, Health and Retirement Study.

**Table 4:** Association of transitions in frailty status with risks of dementia.

Frailty status transition	ELSA and HRS (*n* = 8,353)				
		Model 1		Model 2	
	Events/n	HR (95%CI)	*P*	HR (95%CI)	*P*
Stable robust	65/2928	1 (reference)		1 (reference)	
Robust to pre-frail/frail	40/764	2.50 (1.68–3.70)	<0.001	1.78 (1.18–2.68)	0.006
Stable pre-frail	93/2021	1 (reference)		1 (reference)	
Pre-frail to robust	26/522	1.04 (0.68–1.61)	0.850	1.24 (0.80–1.92)	0.342
Pre-frail to frail	49/502	2.29 (1.62–3.24)	<0.001	1.97 (1.39–2.81)	<0.001
Stable frail	126/1310	1 (reference)		1 (reference)	
Frail to robust/pre-frail	29/306	0.87 (0.58–1.30)	0.498	0.94 (0.62–1.41)	0.754

ELSA, English Longitudinal Study of Aging; HRS, Health and Retirement Study. Model 1 was unadjusted. Model 2 was adjusted for age, sex, education level, marital status, smoking, drinking, social activity, and body mass index.

### 3.4 Associations of total frailty index score and change in frailty index score with dementia

Table 5 presents the association between total FI score and the risk of dementia. Following adjustment for potential confounders, individuals in the highest tertile of total FI were found to have a significantly higher risk of dementia compared to those in the lowest tertile (HR 2.82, 95% CI 2.14–3.72). A significantly elevated risk was also observed among participants in the middle tertile (HR 1.71, 95% CI 1.29–2.27). A test for trend indicated a significant increasing trend in dementia risk with higher levels of total FI (p for trend < 0.001).

**Table 5:** Association of total frailty index score with risks of dementia.

Total frailty index score category	ELSA and HRS (*n* = 8,353)				
		Model 1		Model 2	
	Events/n	HR (95%CI)	*P*	HR (95%CI)	*P*
T1 of total FI	78/3224	1 (reference)		1 (reference)	
T2 of total FI	133/2622	2.21 (1.67–2.92)	<0.001	1.71 (1.29–2.27)	0.005
T3 of total FI	217/2507	4.23 (3.26–5.48)	<0.001	2.82 (2.14–3.72)	<0.001
*p* for trend test		<0.001		<0.001	

Total FI was calculated by the FI at baseline plus the FI at the second survey. T1 was the lower tertile, T2 was the middle tertile, and T3 was the upper tertile. ELSA, English Longitudinal Study of Aging; HRS, Health and Retirement Study. Model 1 was unadjusted. Model 2 was adjusted for age, sex, education level, marital status, smoking, drinking, social activity, and body mass index.

Table 6 displays the association between change in frailty index (ΔFI) score and the risk of dementia. After adjusting for potential confounders, Model 2 revealed that individuals in the highest tertile of ΔFI were at a significantly higher risk of developing dementia than those in the lowest tertile (HR 1.58, 95% CI 1.28–1.94). No statistically significant difference in dementia risk was observed between the middle and lowest tertiles (HR 0.78, 95% CI 0.57–1.07). Additionally, a significant upward trend in dementia risk was observed with increasing ΔFI (p for trend = 0.003). Supplementary Figures S2, S3 illustrate the distributions of total FI and ΔFI, respectively.

**Table 6:** Association of change in frailty index score with risks of dementia.

Change in frailty index score category	ELSA and HRS (*n* = 8,353)				
		Model 1		Model 2	
	Events/n	HR (95%CI)	*P*	HR (95%CI)	*P*
T1 of ΔFI	219/4803	1 (reference)		1 (reference)	
T2 of ΔFI	48/1530	0.69 (0.50–0.94)	0.043	0.78 (0.57–1.07)	0.158
T3 of ΔFI	161/2020	1.90 (1.55–2.32)	<0.001	1.58 (1.28–1.94)	0.002
*p* for trend test		<0.001		0.003	

ΔFI was calculated by the FI at the second survey minus the FI at baseline. T1 was the lower tertile, T2 was the middle tertile, and T3 was the upper tertile. ELSA, English Longitudinal Study of Aging; HRS, Health and Retirement Study. Model 1 was unadjusted. Model 2 was adjusted for age, sex, education level, marital status, smoking, drinking, social activity, and body mass index.

### 3.5 Sensitivity analyses

Sensitivity analyses yielded results largely consistent with the main analyses, supporting the robustness of our findings. (i) After further adjusting for baseline FI, the association between transitions in frailty status and dementia risk remained consistent with the main analyses (Supplementary Table S8). Progression in frailty status continued to be associated with an increased risk of dementia, while no statistically significant association was observed for recovery from frailty. (ii) When frailty status transitions were re-evaluated using data from the third assessment, the increased risk of dementia among robust to pre-frail or frail participants was no longer statistically significant compared to stable robust participants; other results remained consistent with the main analyses (Supplementary Table S9). (iii) Excluding cases of dementia identified during the first year of follow-up, all results were consistent with the main analyses (Supplementary Tables S10–S13). (iv) When alternative FI thresholds were used to define frailty status, results based on Cut-off value 1 remained consistent with the main analyses, whereas under Cut-off value 2, the dementia risk for robust to pre-frail or frail participants was no longer statistically significant compared with stable robust participants (Supplementary Tables S14, S15). (v) Results from competing risk analyses considering dementia and death as competing events were consistent with those of the main analyses (Supplementary Tables S16–S19). (vi) In sex-stratified analyses (Supplementary Tables S20–S23), among women, pre-frail participants did not exhibit a significantly increased risk of dementia compared with robust participants in the baseline frailty status analysis. In the frailty status transition analysis, male robust to pre-frail or frail participants showed no significant difference in dementia risk compared with the stable robust group. In the total FI analysis, female participants in the middle tertile did not have a significantly elevated risk of dementia compared with those in the lowest tertile. All other findings were consistent with the main analyses. (vii) The sensitivity analysis restricted to participants aged ≥65 years yielded results consistent with those of the main analysis (Supplementary Tables S24–S27). (viii) When analyses were conducted separately within HRS and ELSA, the overall patterns of association were consistent with those observed in the combined analyses (Supplementary Tables S28–S37). In both cohorts, baseline frailty and frailty progression were generally associated with increased dementia risk, although some specific transition groups did not reach statistical significance in ELSA. Overall, the direction and general pattern of associations within each cohort supported the primary findings.

## 4 Discussion

Using harmonized data from two prospective cohorts, we examined both baseline frailty status and transitions in frailty in relation to dementia risk. Consistent with prior literature, pre-frail and frail individuals at baseline exhibited a higher risk of dementia compared with robust participants. Extending these findings, we observed that progression in frailty status was consistently associated with elevated dementia risk, whether defined categorically by transition groups or quantitatively by total FI and ΔFI. In contrast, improvement from pre-frail or frail states was not associated with a statistically significant reduction in dementia risk relative to stable counterparts. This asymmetric pattern—where progression increased risk but remission did not confer clear protection—adds nuance to existing evidence and underscores the importance of distinguishing between frailty deterioration and recovery when evaluating dementia risk.

The dynamic nature of frailty has increasingly become a focal point in recent geriatric research. Persistent frailty and transitions to frailty are associated with poorer cognitive performance over time (20). Our findings build upon this by comprehensively quantifying frailty through the FI and demonstrating that frailty progression was independently associated with increased risk of dementia.

However, diverging from previous findings, our results did not demonstrate a statistically significant protective effect of frailty reversal. To our knowledge, only one previous study has examined the association between frailty remission and dementia risk, reporting a lower risk among participants with sustained remission over three consecutive waves (36). Although we also conducted sensitivity analyses using three-wave data to ensure the stability of frailty changes, we did not observe a significant reduction in dementia risk among participants who experienced frailty remission relative to those with stable frailty status. This discrepancy may be partly explained by differences in frailty assessment tools. Their study assessed frailty using the Fried frailty phenotype, which includes five physical components: exhaustion, low physical activity, shrinking, weakness, and slowness (37). In contrast, our study employed the cumulative deficit model, evaluating frailty by calculating the FI. The Fried phenotype focuses on clinically observable physical decline, whereas the FI provides a more comprehensive assessment by incorporating chronic diseases, symptoms, functional impairments, and other health deficits. While previous literature indicates that both frailty measures may have similar predictive value for cognitive outcomes (38), our results did not support a significant association between frailty remission, as defined by FI, and reduced dementia risk.

To further explore potential explanations for findings, we examined recovery proportions for each FI component (Supplementary Table S38). We found that frailty remission predominantly occurred in physical function domains (items 9–10 and 25–34). This suggests that although physical function recovery may occur, it may not be sufficient to significantly alter long-term dementia risk. This suggests that the absence of a statistically significant reduction in dementia risk among participants who experienced frailty remission may be partly related to the specific FI components in which improvement occurred. Improvements in FI components outside the physical function domain (e.g., chronic conditions) may have different implications for cognitive outcomes. Therefore, domain-specific trajectories of FI components warrant further investigation. Additionally, the absence of a statistically significant reduction in dementia risk among participants who experienced frailty improvement is consistent with the possibility that the timing and extent of deficit accumulation may influence the potential for subsequent risk reduction. Dementia is widely recognized as a progressive clinical syndrome resulting from the accumulation of neurodegenerative and vascular pathology that may develop over an extended period prior to clinical diagnosis (39). If such pathological processes have already advanced prior to the observed improvement in frailty status, subsequent changes in overall frailty may not translate into a measurable reduction in dementia during the available follow-up period. This interpretation is presented as a hypothesis and warrants further investigation in studies with longer follow-up and more detailed clinical phenotyping.

Our study provides new evidence linking frailty to dementia by emphasizing its dynamic nature and relevance to dementia risk. Our findings emphasize the importance of viewing frailty as a dynamic and modifiable condition rather than a static baseline characteristic. While previous studies have focused on the adverse impact of baseline frailty on dementia, our results further demonstrate that progression in frailty status is significantly associated with an increased risk of developing dementia. This underscores the need for early identification and monitoring of frailty progression in older adults as a component of dementia prevention strategies. Moreover, the absence of a significant reduction in dementia risk among participants who recovered from pre-frail or frail status suggests that frailty reversal may not consistently confer cognitive benefits. This may be attributable to inconsistencies in frailty assessment tools, variations in the timing and severity of frailty, or underlying biological mechanisms. Given the heterogeneity in frailty assessment tools and study designs, our findings warrant replication in other cohorts to confirm their stability and generalizability. Overall, these results highlight the potential of targeted interventions aimed at preventing frailty progression as a promising approach to mitigating dementia risk in aging populations.

This study has several strengths. First, this is the initial analysis exploring how transitions in frailty status relate to dementia risk, offering a novel perspective on the impact of dynamic frailty transitions. Second, it combined two large prospective cohorts and aligned follow-up periods across datasets to ensure temporal consistency, thereby enhancing the representativeness of the study population. The large integrated sample size also increased the statistical power of the analyses. Finally, multiple sensitivity analyses were conducted to confirm the robustness of the finding.

This study has several limitations. First, the identification of dementia was based on self-reported physician diagnoses, which may have led to an underestimation of the true prevalence in the study population. Second, dementia in this study was treated as a composite outcome, encompassing all-cause dementia. Although evidence suggests that mixed dementia becomes the predominant subtype with advancing age (40), further research is warranted to explore the association between transitions in frailty status and the risk of specific dementia subtypes. Third, although we adjusted for a wide range of covariates, residual confounding from unmeasured factors cannot be fully excluded. Genetic susceptibility and detailed clinical or biological markers were not available in the datasets and may influence both frailty transitions and dementia risk. Fourth, in ELSA, death information was only available up to Wave 6, whereas dementia follow-up continued through Wave 9. Participants without dementia were censored at their last observed interview year. Because deaths occurring between Waves 7 and 9 could not be distinguished from other forms of loss to follow-up, mortality during this interval may have been treated as non-informative censoring. Given the association between frailty and mortality (41), incomplete death ascertainment may have introduced informative censoring and potentially attenuated the estimated associations between frailty progression and incident dementia.

In conclusion, progression in frailty status is significantly associated with an increased risk of dementia, underscoring the value of early identification and intervention to prevent or delay cognitive decline. In contrast, recovery from pre-frail or frail states was not significantly associated with a reduced risk of dementia in our study. However, given the limited understanding of the biological and functional mechanisms underlying frailty remission, further research is warranted to clarify whether frailty reversal can contribute to dementia risk reduction and to assess its potential as a viable intervention target.

## Data Availability

Publicly available datasets were analyzed in this study. This data can be accessed through the official websites of English Longitudinal Study of Aging (https://www.elsa-project.ac.uk/) and Health and Retirement Study (https://hrs.isr.umich.edu/).

## Footnotes

1
https://dagitty.net/dags.html
