# Supplementary material for: Association of transitions in frailty with dementia risk: findings from two longitudinal cohort studies
Source: Front Med (Lausanne). 2026 Mar 26;13:1782916. doi: 10.3389/fmed.2026.1782916 (PMC13062179; doi:10.3389/fmed.2026.1782916)
Supplement: Supplementary file 1 [file Data_Sheet_1.docx]

Supplementary Material

## Supplementary Figures


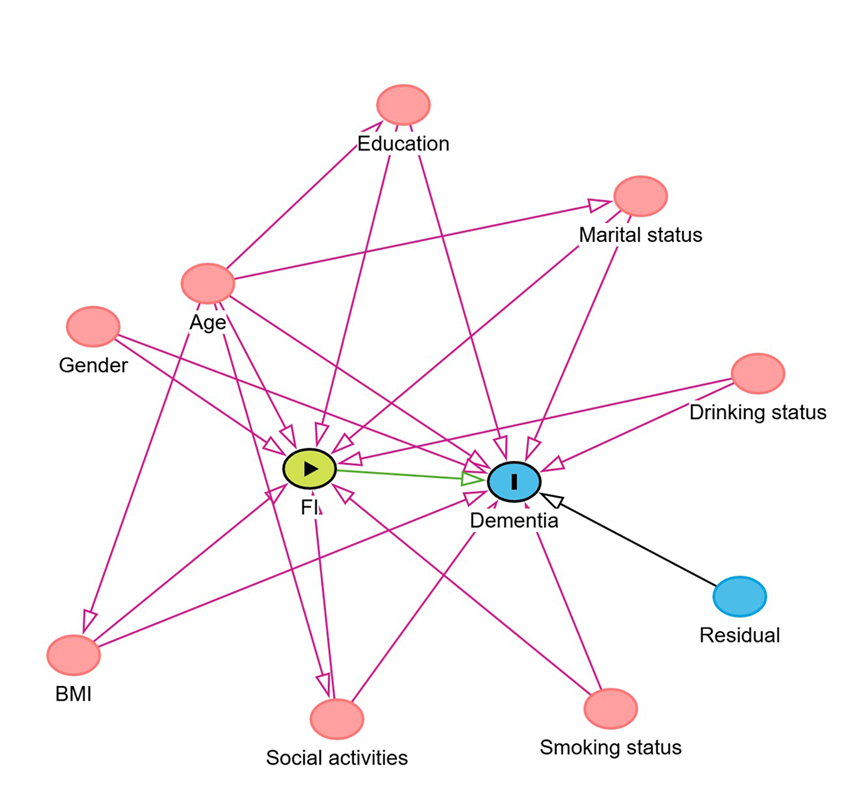


**Figure S1.** Directed acyclic graph.


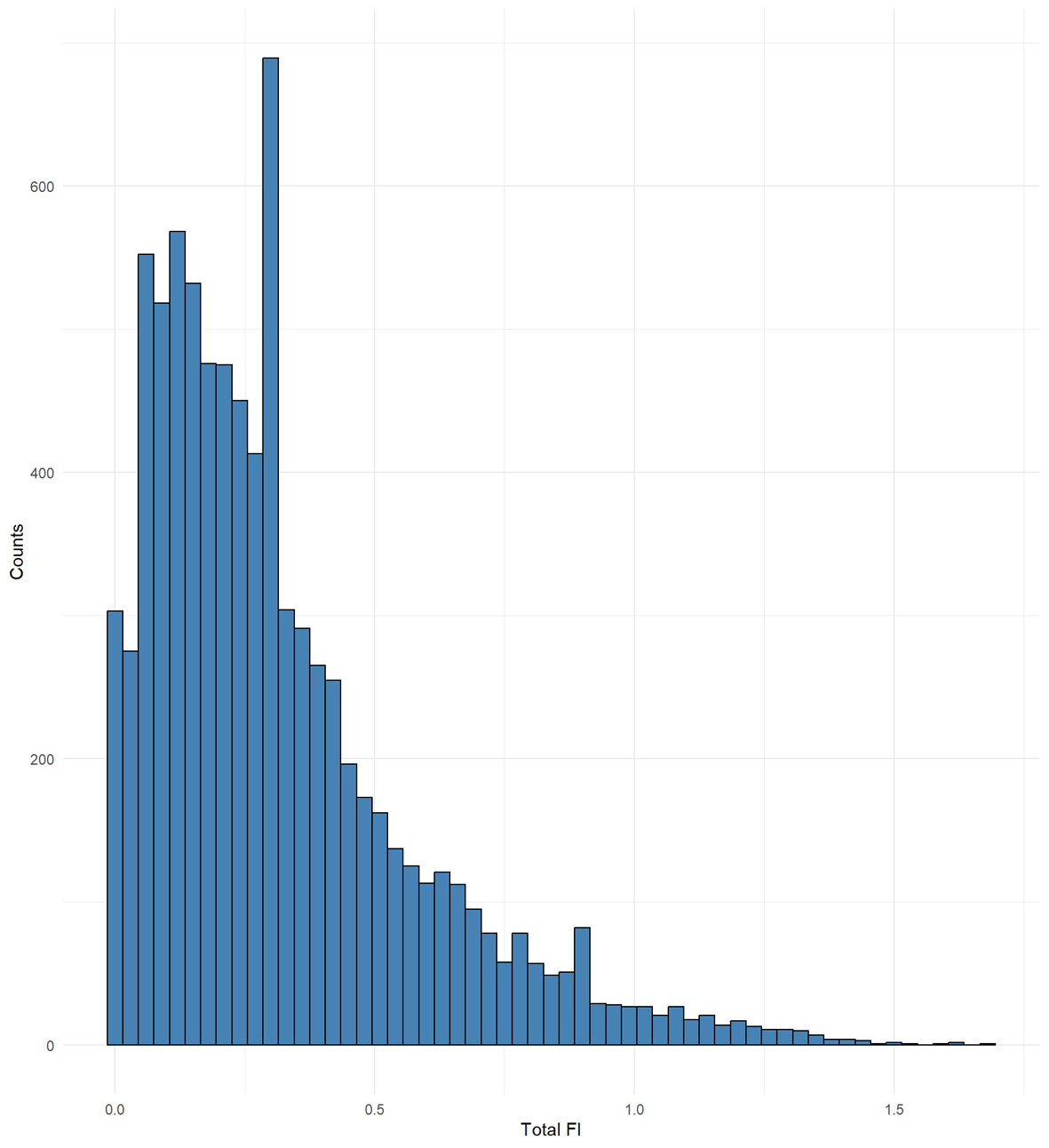


**Figure S2.** Distribution of counts for total frailty index score.


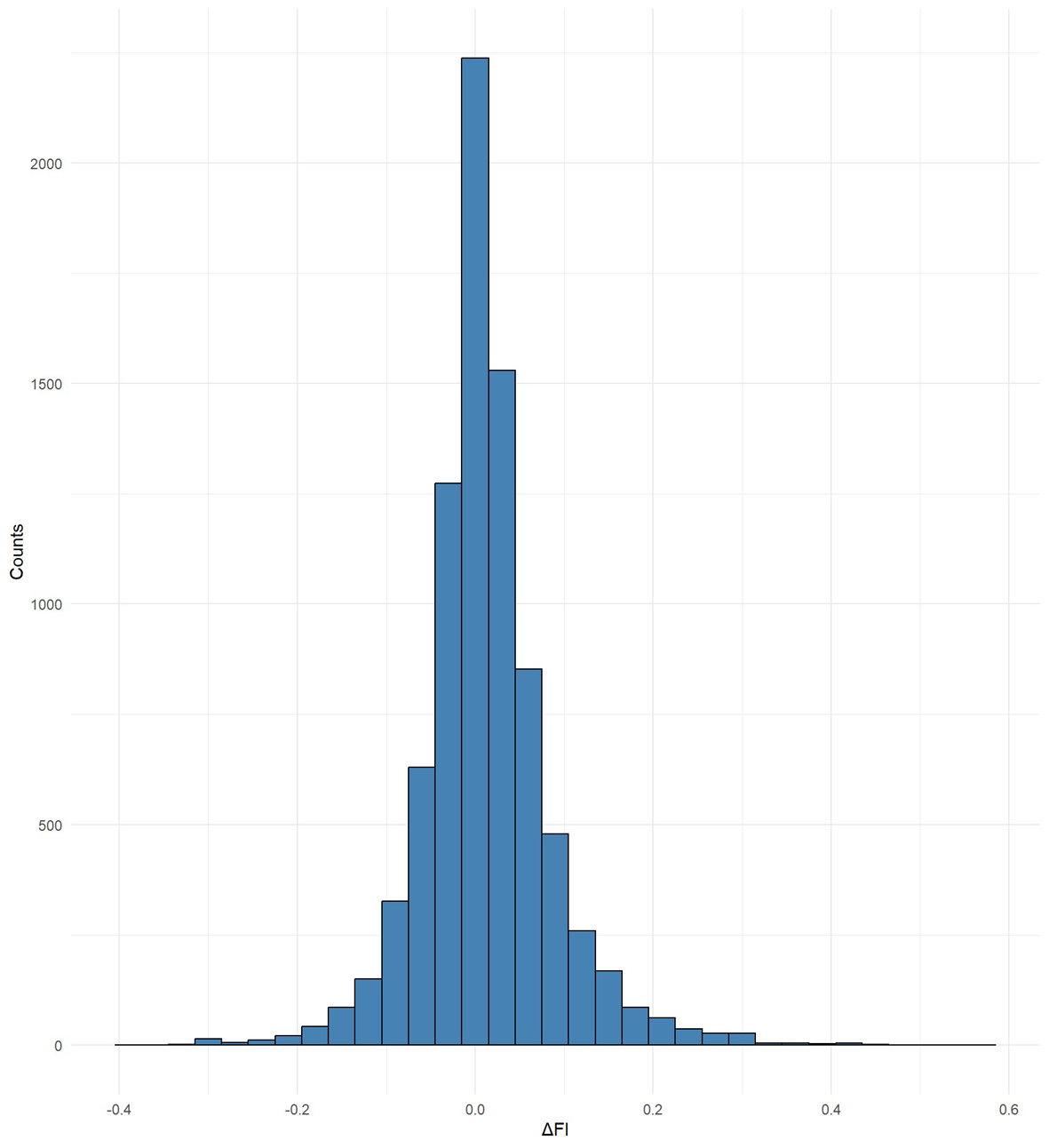


**Figure S3.** Distribution of counts for the change in frailty index score.
